# Adult Neuroblastoma Complicated by Increased Intracranial Pressure: A Case Report and Review of the Literature

**DOI:** 10.1155/2014/341980

**Published:** 2014-09-25

**Authors:** Patrick L. Stevens, Douglas B. Johnson, Mary Ann Thompson, Vicki L. Keedy, Haydar A. Frangoul, Kristen M. Snyder

**Affiliations:** ^1^Divisions of Hematology and Oncology, Department of Medicine, Monroe Carell Jr. Children's Hospital at Vanderbilt, Vanderbilt University Medical Center, Nashville, TN, USA; ^2^Department of Pathology, Microbiology, and Immunology, Monroe Carell Jr. Children's Hospital at Vanderbilt, Vanderbilt University Medical Center, Nashville, TN, USA; ^3^Division of Pediatric Hematology and Oncology, Department of Pediatrics, Monroe Carell Jr. Children's Hospital at Vanderbilt, Vanderbilt University Medical Center 2220 Pierce Avenue, 397 PRB, Nashville, TN 37232-6310, USA

## Abstract

Neuroblastoma is the third most commonly occurring malignancy of the pediatric population, although it is extremely rare in the adult population. In adults, neuroblastoma is often metastatic and portends an extremely poor overall survival. Our case report documents metastatic neuroblastoma occurring in a healthy 29-year-old woman whose course was complicated by an unusual presentation of elevated intracranial pressures. The patient was treated with systemic chemotherapy, I^131^ metaiodobenzylguanidine (MIBG) radiotherapy, and autologous stem cell transplant (SCT). Unfortunately the patient's response to therapy was limited and she subsequently died. We aim to review neuroblastoma in the context of increased intracranial pressure and the limited data of neuroblastoma occurring in the adult population, along with proposed treatment options.

## 1. Introduction

Neuroblastoma is the most commonly occurring extracranial solid tumor diagnosed in the pediatric population [1, 2]. Ninety percent of diagnoses occur in patients less than 4 years of age [3, 4]. Furthermore, outcomes in adults are significantly worse than those in children.

Neuroblastoma is a disease of the sympaticoadrenal neural crest cells and may occur in any part of the sympathetic nervous system. Symptoms of the disease vary and are dependent on tumor location, presence of metastatic disease, and paraneoplastic syndromes. Typical sites of involvement include the adrenal medulla, neck, chest, and pelvis [2]. Nearly 50% of patients present with metastasis to distant sites. Up to 5% of patients are diagnosed with paraspinal tumors which present with signs of cord compression [5]. Involvement of the bone marrow can lead to complete hematopoietic failure and pancytopenia [6]. Common paraneoplastic syndromes in neuroblastoma include secretion of vasoactive intestinal peptide resulting in voluminous watery diarrhea and opsoclonus myoclonus syndrome, the “dancing eyes dancing feet” syndrome [7, 8]. We herein present a case of an adult patient diagnosed with metastatic neuroblastoma complicated by severe increases in intracranial pressure despite no evidence of central nervous system disease.

## 2. Case Report

**Figure 1:**
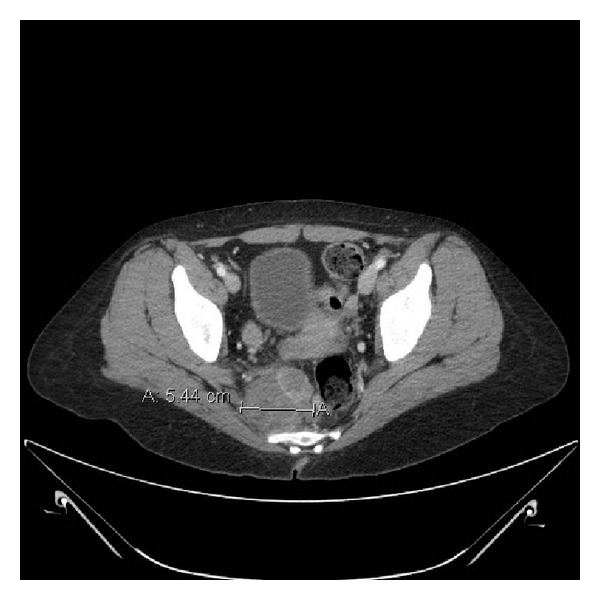
CT scan of the pelvis at the level of the iliac crest reveals a 5.4 cm soft tissue mass. Bones of the pelvis demonstrate abnormal texture.

A previously healthy 29-year-old female originally presented with blurred vision, headaches, paresthesias of the fingers, and several days of fatigue. A complete blood count revealed a white blood cell count of 4,000/*μ*L, hematocrit of 18%, platelets of 32,000/*μ*L immature myeloid cells, and nucleated red cells consistent with a leukoerythroblastic smear. Initial computed tomography (CT) scans of the head, chest, abdomen, and pelvis was notable for a 5 cm soft tissue mass in the right pelvis, anterior to the sacrum with a lytic lesion in the left iliac crest (Figure 1). Further imaging with magnetic resonance imaging (MRI) of the brain revealed a diffuse dural thickening without intracranial lesions. A CT guided biopsy of the pelvic mass was initially diagnosed as a ganglioneuroma. Further testing was notable for an elevated 24-hour urine norepinephrine level of 694 *μ*g (nl 0–135 *μ*g).

**Figure 2:**
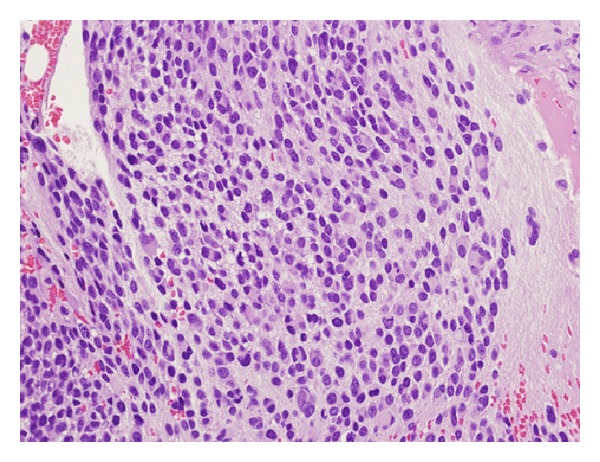
The H&E stained section of the bone marrow biopsy demonstrates extensive involvement by neuroblastoma with a background of neuropil. Magnification is 400x.

**Figure 3:**
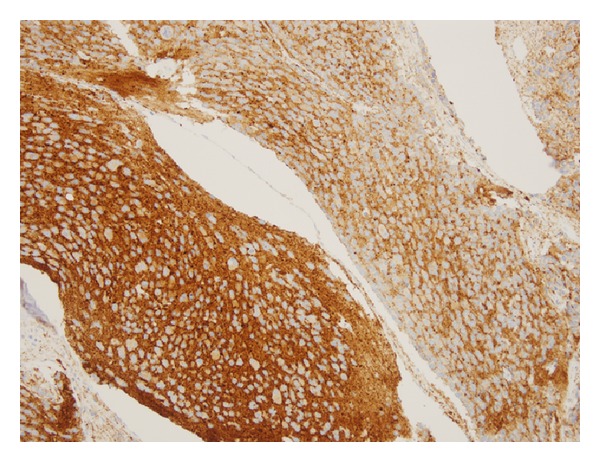
Immunohistochemistry of a paraffin section of the bone marrow biopsy using an antibody directed against synaptophysin demonstrates dense punctate cytoplasmic staining of the tumor cells, including staining of the neuropil. Magnification is 200x.

The patient initially received chemotherapy at an outside institution with cisplatin 80 mg/m^2^ on day 1 and etoposide 80 mg/m^2^/day on days 1–3 for the presumed diagnosis of metastatic neuroendocrine carcinoma. However, bone marrow evaluation revealed 80% marrow involvement of neuroblastoma with little evidence for differentiation. At this point, she was referred to our institution for further evaluation. The outside pelvic mass biopsy was reviewed. Taking into account imaging and bone marrow findings, the pelvic mass was best classified as a composite (nodular) ganglioneuroblastoma. A repeat bone marrow biopsy performed at our institution demonstrated extensive involvement by metastatic neuroblastoma (Figure 2). The majority of the tumor cells were immature with a background of neuropil, with a small proportion of cells showing ganglionic differentiation. Immunohistochemical studies demonstrated strong cytoplasmic staining for tyrosine hydroxylase and synaptophysin in the tumor cells (Figure 3).

At our initial visit, the patient continued to complain of visual changes and headache. Immediate ophthalmology evaluation revealed significant bilateral papilledema. A lumbar puncture was performed with opening pressure of >55 cm H_2_O; cerebrospinal fluid (CSF) cytology was negative for involvement by neuroblastoma.

Brain MRI was notable for diffuse dural enhancement and thickening and restricted diffusion within the calvarium concerning for metastatic disease to the dura and calvarium, with absence of normal flow in the transverse sinuses secondary to dural compression. Intravenous acetazolamide and methylprednisolone were administered for elevated intracranial pressure with initial improvement in her visual symptoms. Her serum vanillylmandelic acid and homovanillic acid prior to subsequent treatment were 243 ng/mL (nl < 20) and 93 ng/mL (nl < 30), respectively. She commenced antineoplastic therapy with topotecan 1.2 mg/m²/dose and cyclophosphamide 400 mg/m²/dose on days 1–5. The patient was discharged post-therapy with a stable ophthalmologic exam.

She then received her second cycle of cyclophosphamide and topotecan despite thrombocytopenia (18,000/*μ*L), given her degree of bone marrow infiltration with neuroblastoma. Following cycle two, she developed worsening headaches and vision changes, with MRI findings of increased dural enhancement and compression of the sagittal sinuses without development of venous thrombosis. A lumbar drain, later converted to a ventriculoperitoneal (VP) shunt, provided minimal symptomatic relief.

Despite medical therapy with acetazolamide and surgical placement of a VP shunt, her symptoms worsened, ultimately resulting in return of headaches and inability to see light from the left eye with minimal light appreciation in the right. Ophthalmology performed bilateral nerve sheath fenestration which allowed for light perception in both eyes. At this point the patient had received no additional myelosuppressive chemotherapy for approximately 8 weeks given these complications. Restaging following cycle two demonstrated an increase in the size of her pelvic mass to 7.3 cm × 5.6 cm and persistent bone marrow involvement.

**Figure 4:**
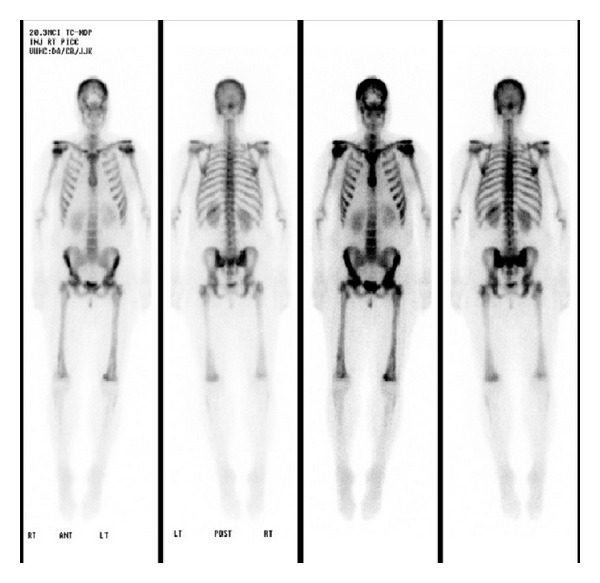
Diffuse metastatic involvement of the osseous skeleton consistent with known diagnosis of neuroblastoma with a large right pelvic mass. The two left most panels represent pre-MIBG treatment. The two right-most panels represent images obtained 25 days following therapeutic MIBG.

She was then treated with therapeutic I^131^-MIBG (Figure 4), which was well tolerated. Following MIBG therapy, she was able to discontinue all narcotics and became symptom free. Subsequent MIBG imaging obtained 25 days after therapy revealed persistent disease (Figure 4). Her neurologic symptoms returned 40 days after therapy and a decision was made by the patient and her family to focus on comfort care. She died 60 days following MIBG therapy.

## 3. Discussion

Neuroblastoma is an embryonal tumor of the sympathetic nervous system and is the third most common childhood cancer. However, this diagnosis is extremely rare in adults, with less than 100 cases reported in the literature. Our patient presented with metastatic neuroblastoma complicated by a likely paraneoplastic syndrome associated with severely increased intracranial pressure.

Adults with metastatic neuroblastoma typically have a very poor prognosis [9]. Patients older than 18 months at the time of diagnosis typically present with unfavorable risk features including n-myc amplification. Such high-risk disease is treated with a combination of myelosuppressive chemotherapy and autologous stem cell transplant, followed by anti-GD-2 therapy and retinoic acid differentiation therapy. This combination of therapy has provided event free survival outcomes of 86% at 2 years [10]. The best outcomes occur in patients under 1 year of age who are diagnosed with early stage or stage 4S neuroblastoma but are without high-risk features such as n-myc amplification. These patients can often be monitored closely for disease resolution without therapy [11].

Disease presentation is often similar in adults and children, though several differences have been described. Disease progression is often more indolent in adults compared with children. A significant minority has a slowly progressive disease or recurrent relapses over a period of years. Tumors are of the sympathetic nerve chain and thus locations of primary tumors occur similarly in adults and children [12]. Notably, n-myc amplification is common in the high-risk pediatric population but appears very rarely in adults [13].

There is no consensus on the treatment for neuroblastoma in adults patients. Surgery and radiation therapy may be sufficient for localized disease. For metastatic disease, combination chemotherapy with or without radiation therapy may be fairly effective. Based on a report by Kushner et al. of nine adolescents and adults treated with a combination of doxorubicin, vincristine, cyclophosphamide, cisplatin, and etoposide, six experienced at least partial responses, three of which had a complete response or very good partial response [13]. Consolidation treatment with high dose chemotherapy and autologous stem cell transplant has been shown to improve survival in children and is an option in adults as well [14]. However, in the salvage setting, results have been unimpressive with complete response or partial response (CR/PR) occurring in only 19% of primary refractory disease and 52% of secondary refractory disease [15]. Novel treatments including those targeting anaplastic lymphoma kinase (ALK) mutations, tumor associated disialoganglioside antigen GD3 and targeted radiotherapy will also likely have a role in adults [16–18].

Paraneoplastic syndromes have been described in patients with neuroblastoma. Most commonly described is the opsoclonus-myoclonus syndrome which occurs in 1–3% of patients. This syndrome is characterized by rapid, conjugate, multidirectional eye movements as well as myoclonus and ataxia [19]. In addition, it carries a risk of long-term neurological sequelae [20]. Additionally described neurologic paraneoplastic phenomena include Lambert-Eaton myasthenic syndrome, anti-Hu syndrome, ataxia, and idiopathic bilateral ptosis [21–24]. Nonneurologic paraneoplastic syndromes include secretory diarrhea from vasoactive intestinal peptide (VIP) secretion, Cushing's syndrome, hypercalcemia, and the syndrome of inappropriate antidiuretic hormone (SIADH) [12, 23, 25].

While our patient's increased intracranial pressure may have been due to a paraneoplastic phenomenon, disease in the central nervous system (CNS) itself could not be excluded. Though CNS involvement was suggested by dural enhancement seen on MRI, repeat cerebrospinal fluid (CSF) sampling over a period of months was negative by cytology. CNS involvement is very uncommon in neuroblastoma, with a rate of 0.6% in one large case series [26]. When the CNS involvement is present, dural enhancement may occasionally be the only imaging abnormality, though often dural nodularity is additionally present [27]. A single case report describes paraneoplastic papilledema in an adult with neuroblastoma, although symptoms were limited to a moderate headache [28]. In that case, opening pressure was not elevated.

Our patient's primary disease appeared to arise from a lesion in the pelvis. In a series of 25 adult/adolescent patients, 16% had disease occurring in the pelvis, while 68% arose in the retroperitoneum or adrenal glands [13].

This case represents the presentation of a common disease in an uncommon patient population. The majority of adult oncologists will not experience caring for a patient with neuroblastoma; however, this diagnosis should at least be considered in certain cases. Neuroblastoma may occur in similar sites to neuroendocrine tumors and should be considered particularly in patients of adolescent age and those with neurologic paraneoplastic symptoms. Furthermore, due to the rarity of these diseases, diagnostic evaluation by an experienced pathologist is warranted to distinguish between neuroendocrine tumors and the rare case of adult neuroblastoma.

## Abbreviations

ALK: Anaplastic lymphoma kinase
CNS: Central nervous system
CSF: Cerebrospinal fluid
CT: Computed tomography
MRI: Magnetic resonance imaging
NSE: Neuron-specific enolase
SIADH: Syndrome of inappropriate diuretic hormone
VIP: Vasoactive intestinal peptide
VP: Ventriculoperitoneal.
